# Winter tapping of cold-tolerant rubber clone Yunyan 77-4: a viable strategy to reschedule the production season and capture potential market premiums in China

**DOI:** 10.3389/fpls.2026.1791687

**Published:** 2026-04-23

**Authors:** Yifan Li, Yaowen Yang, Qianghao Zhang, Mingqian Li, Haining He, Yanlin Xiong, Xiaojun Gao, Yanqin Yin, Jin Liu

**Affiliations:** 1 Yunnan Key Laboratory of Sustainable Utilization Research on Rubber Tree, Xishuangbanna, China; 2 Yunnan Institute of Tropical Crops, Xishuangbanna, China

**Keywords:** cold-resistant, *Hevea brasiliensis*, rubber production, rubber tree, winter tapping, Yunyan 77-4

## Abstract

Natural rubber production in China is constrained by a relatively cold climate, a short tapping season, and concentrated rainfall, leading to lower yields than that in tropical nations. To strengthen the domestic rubber supply and enhance international competitiveness, novel cultivation and tapping approaches must be explored to expand planting areas and unlock yield potential. This study employed the cold-tolerant clone Yunyan 77–4 in winter tapping trials to establish a theoretical and practical basis for optimizing the tapping season and expanding winter tapping areas. The results demonstrated that: 1) Winter tapping effectively avoids interference from the rainy season, with rainfall during this period being 65.07% lower and rainy days being 51.61% fewer compared to the traditional tapping season, thereby lowering labor intensity and improving operational efficiency. 2) Under the same operational duration, winter tapping showed no significant differences in fresh latex yield, theoretical dry rubber yield, or yield per unit area compared to the traditional tapping. 3) The tapping technique used in winter tapping was largely consistent with that of traditional tapping, with no significant differences in incidence rate of tapping panel dryness (TPD), incidence index of TPD, tapping depth, and bark consumption. Additionally, no significant difference in tree circumference was observed between the two tapping seasons, indicating that no unreasonably tapping practices were employed to enhance rubber yield. 4) The implementation of winter tapping aligns production with periods of historically higher futures prices, suggesting a potential for capturing market premiums of 8.86–20.81% under normal seasonal conditions. In conclusion, winter tapping is technically feasible and economically advantageous.

## 1 Introduction

Natural rubber, as a critical industrial raw material, is indispensable for the national economy and strategic security sectors. It is widely used in core industries such as transportation, healthcare, aerospace, military manufacturing, and construction, holding significant strategic value and economic importance (Liu et al., 2020; Weerathamrongsak and Wongsurawat, 2013). The rubber tree (*Hevea brasiliensis*), as a primary source species for natural rubber, dominates the global natural rubber industry due to its biological characteristics, which allow its latex system to continuously secrete high-purity latex (Bai et al., 2024; Qi et al., 2024). However, the growth of rubber trees is highly dependent on specific climatic conditions, requiring sufficient heat, precipitation, appropriate altitude, and a suitable temperature range (Huang et al., 2022; Rao and Vijayakumar, 1992; Ray et al., 2016). These ecological constraints have significantly limited rubber cultivation in China, posing long-term risks to the stability of the domestic supply.

Owing to geographical latitude and climatic restrictions, natural rubber plantations in China are primarily distributed in the northern tropical margins between 18°N and 24°N, covering provinces such as Yunnan, Hainan, Fujian, Guangdong, and Guangxi (Xu, 2006; Priyadarshan et al., 2005; Wang et al., 2023). Compared to major Southeast Asian producing countries such as Thailand, Indonesia, and Vietnam, China’s rubber-growing regions are situated at higher latitudes with lower annual average temperatures, resulting in a significantly shorter tapping season (typically only 7–9 months). This limitation severely constrains domestic natural rubber production capacity and leads to a substantial deficit in self-sufficiency. According to statistics, China accounts for only approximately 6% of global natural rubber production, while requiring imports of approximately 7 million tons of natural and synthetic rubber annually to meet domestic industrial demand (National Bureau of Statistics of China, 2024). This heavy reliance on the international market introduces significant risks to the rubber supply chain.

In addition, after nearly seven decades of development, China’s natural rubber industry continues to face multiple structural challenges: (1) Sustained low economic returns. Since 2011, natural rubber prices have fallen sharply from over CNY 40,000 per ton to approximately CNY 12,000 per ton by 2022, severely dampening farmers’ enthusiasm for production and resulting in a pronounced mismatch between labor intensity and income (Liu et al., 2024). (2) Low production efficiency. The per-unit yield in Hainan is only approximately 60% of that in major Southeast Asian regions, with natural conditions directly limiting yield improvements (Wang and Ke, 2014). (3) Significant environmental constraints. Limited suitable cultivation areas are frequently affected by meteorological disasters, such as low temperatures and typhoons, leading to unstable production (Shen et al., 2025). (4) Pronounced labor shortages. The demanding working conditions and disruptive circadian rhythms associated with tapping have led to a severe outflow of tappers, further hindering the sustainable development of the industry (Jin et al., 2021). Therefore, improving yield per unit area, reducing labor intensity, expanding suitable cultivation areas, and increasing farmers’ income have become urgent priorities for the industry.

In response to these challenges, Chinese researchers have made significant breakthroughs over decades of effort in cold-tolerant breeding and cultivation techniques, successfully developing a series of rubber tree germplasms with enhanced cold resistance and environmental adaptability. Notable examples include CATAS7-33-97 (also known as ‘Reyan7-33-97’) (Chao et al., 2023), ‘Yunyan 77-4’ (Mao et al., 2023), ‘Yunyan 77-2’ (Li, 2005), ‘Yunyan 80-1983’ (Li et al., 2024), and ‘Dafeng 95’ (Liu et al., 1996). Among these, the triploid clone ‘Yunyan 77-4’ stands out for its rapid growth, excellent cold tolerance, and stable latex production (Li, 2005; Xu et al., 2025). This clone has passed national certification and is listed as a widely promoted clone, becoming the dominant clone in Yunnan’s rubber plantations. Compared to the traditional clone GT1, ‘Yunyan 77-4’ demonstrates superior cold resistance, latex yield, and overall stress tolerance (Qi et al., 2025), offering potential for expanding rubber cultivation to higher latitudes and enabling off-season tapping.

Conventionally, rubber tapping in China is concentrated in the warm rainy season from April to November, with a winter dormancy period that allows tree recovery and nutrient accumulation. However, in major producing areas such as Yunnan, rainfall is highly unevenly distributed, overlapping significantly with the tapping season. Frequent rainfall not only disrupts tapping operations and reduces latex yield and quality, but also increases labor intensity and management costs, thereby constraining economic efficiency. In recent years, with the development and promotion of cold-tolerant varieties, winter tapping has transitioned from a theoretical concept to a practical possibility. This innovative approach has the potential to optimize the annual tapping period, increase yield per plant, avoid rainy season disruptions, and significantly improve tapping efficiency and production stability.

Based on the aforementioned industrial context and research requirements, we hypothesized that the cold-tolerant clone ‘Yunyan 77-4’ possesses the physiological traits necessary to sustain latex production and tree health during the winter period. The implementation of a structured winter tapping system for the cold-tolerant clone ‘Yunyan 77-4’ would (1) possess a substantial latex yield, (2) have no significant impact on tapping panel dryness (TPD) incidence and rubber tree growth, and (3) offer potential for capturing market premiums. Therefore, this study employed the cold-tolerant clone ‘Yunyan 77-4’ in a three-year comparative experiment in Yunnan, contrasting traditional tapping with winter tapping. This study systematically measured core indicators, including latex yield and its components, the incidence rate and incidence index of TPD, consistency of tapping techniques, and potential economic benefits. The practical application value of winter tapping in actual production was comprehensively evaluated from three dimensions: technical feasibility, production stability, and economic rationality. The core novelty of this study lies in being the first to systematically validate the feasibility of combining a specific cold-tolerant clone ‘Yunyan 77-4’ with a structured winter tapping system as a viable alternative production strategy in China’s northern marginal rubber-growing areas. While previous studies have documented the cold tolerance of clones such as ‘Yunyan 77-4’, their application in formal winter tapping systems, which fundamentally reshape the annual production cycle, has remained unexplored. This study provides a comprehensive, multi-dimensional assessment of this practice, evaluating yield, tapping technique, tree growth, TPD incidence, and economic benefits. This represents a paradigm shift from merely documenting cold tolerance to actively leveraging cold-tolerant traits for productive purposes during the traditional dormancy period in a specific marginal region. The findings offer a scientific basis for optimizing tapping systems in China and have significant reference value for other northern marginal rubber-growing areas facing similar climatic constraints and short tapping seasons. Ultimately, this research aims to enhance domestic natural rubber self-sufficiency, reduce external dependency, and promote the sustainable development of the natural rubber industry.

## 2 Materials and methods

### 2.1 Site description

The experimental site was located at the Junhong Experimental Site of the Yunnan Institute of Tropical Crops (22°2’10.61”N, 100°46’55.52”E). This site serves as a national key experimental base for rubber. The experimental materials were planted on mountainous terrain at an elevation of 500–600 m, predominantly on east-facing slopes with gradients ranging from 3° to 7°. The site exhibits a tropical rainforest climate, characterized by consistently high temperatures and abundant year-round rainfall. The annual average temperature ranges from 18.6–21.9 °C, with annual precipitation ranging from 1200 to 1700 mm. The annual sunshine hours average 1800 to 2300 hours, relative humidity ranges from 80% to 86%, and the annual accumulated temperature ≥10 °C is 5062 to 8000 °C. The region exhibits distinct rainy (May to October) and dry (November to April) seasons (Yuan et al., 2025).

### 2.2 Experimental methods and design

The rubber tree clone selected was Yunyan 77-4, which was developed through artificial hybridization of the high-yielding, cold-tolerant GT1 and PR107 parents to create breeding material exhibiting hybrid vigor in yield and resistance traits (Xiao, 1997). In 2012, grafted seedlings were planted as experimental materials at a density of 375–450 trees per hectare, with 2.5–3 m inter-tree spacing and 8–12 m inter-row spacing. The experiment employed randomized block design with two treatments: Traditional Tapping (TT) and Winter Tapping (WT). Each treatment had four replicates, totaling eight experimental plots. Each replicate was demarcated into plots, each approximately 0.67–1.00 hm², with a total experimental area of 6.61 hm². The number of rubber trees per replicate was as follows: TT 1 (349 trees), TT 2 (324 trees), TT 3 (356 trees), TT 4 (413 trees), WT 1 (288 trees), WT 2 (355 trees), WT 3 (336 trees), and WT 4 (353 trees). A total of 2,774 rubber trees were monitored during the experiment. The first tapping of experimental trees commenced in 2021, with the tapping criterion set at a tree circumference exceeding 50 cm. Due to growth variations among individual trees, some rubber trees did not meet the tapping criteria, delaying the experiment by one year. Although a small number of trees still failed to meet the standards in 2022, the experiment was formally initiated that year to prevent further interference from tapping years on the outcomes.

Traditional tapping: Rubber is tapped from April to November, with tapping suspended from December to March of the following year. Winter tapping: Rubber is tapped from September to April of the following year, with tapping suspended from May to August to avoid high temperatures and rainy season weather. The trial period runs from April 2022 to April 2025. Tapping began daily at 2:00 AM, and latex collection commenced at 9:00 AM.

### 2.3 Experimental management

All rubber trees were managed with uniform fertilization and disease control practices. The fertilization regimen is as follows: fertilization occurs three times annually. In May and September, 0.72 kg/tree and 0.48 kg/tree of compound fertilizer (N:P:K = 15:15:15) were applied, respectively. In November, 5 kg/tree of organic fertilizer (organic matter content >30% on a dry weight basis) was applied, resulting in a total annual application of 6.2 kg/tree. In May, 15–20 cm deep trenches were dug for band application. All fertilizers were spread evenly in the trenches and then covered with 5–10 cm of soil immediately after application.

Powdery mildew control begins in February, during bud break and leaf expansion. Sulfur powder was applied at a rate of 22.5 kg/ha. Multiple sprays are applied to severely affected areas. The assessment criteria were as follows:

1. When disease onset occurs before 30% leaf expansion, severe infection is likely that year.
2. Before 60% leaf expansion, moderate infection is likely to occur.
3. Before 70% leaf expansion, prompt control measures should be implemented.
4. After 70% leaf expansion (if the weather conditions are favorable), spraying is generally unnecessary.

Throughout the three-year experimental period (2022–2024), no abnormal incidence of powdery mildew was observed in either treatment.

### 2.4 Data collection

#### 2.4.1 Meteorological data

This study conducted daily observations of temperature and precipitation throughout the entire experimental period to compare and analyze climatic differences between winter tapping and traditional tapping. Meteorological data were collected using a HOBO U30 NRC portable mini automatic weather station (Onset Computer Corp., MA, USA).

#### 2.4.2 Determination of latex yield and dry rubber content

The rubber trees were tapped according to the S/2 d/4 tapping pattern. After latex flow ceased, fresh latex was collected from all trees in each replicate. The collected latex was transported to the rubber processing room for yield and dry rubber content (DRC) determination. Rubber latex yield was measured as follows: the weight of fresh latex collected during each tapping session was recorded. Upon completion of the entire tapping period, the total fresh latex weight was calculated for each replicate. DRC refers to the percentage of rubber hydrocarbons in the total latex weight. During each fresh latex collection, a 30mL sample was taken from each replicate. DRC was measured using a dry content analyzer (DH925D Microwave Latex Tester) and recorded.

(1) $$\begin{array}{l} Theoretical\;dry\;rubber\;yield\;(kg)=Fresh\;latex\;yield\\ \times Average\;DRC\; \end{array}$$

(2) $$\begin{array}{l} Yield\;per\;plant\;(kg/plant)=Theoretical\;dry\;rubber\;yield/\\ Number\;of\;effective\;tapping\;trees \end{array}$$

(3) $$\begin{array}{l} Yield\;per\;plant\;per\;tapping\;(g/plant/cut)=\\ Yield\;per\;plant\;/Number\;of\;rubber\;tapping\times 100 \end{array}$$

(4) $$\begin{array}{l} Yield\;per\;unit\;area\;(kg/hm^{2})=Theoretical\;dry\;rubber\;yield\\ /Trial\;plot\;area\;(hm^{2}) \end{array}$$

#### 2.4.3 Rubber tree circumference

According to rubber industry standards, bud-grafted rubber trees must reach a circumference of 50 cm at 100 cm above ground level, or superior seedling rubber trees must reach 50 cm at 50 cm above ground level before formal tapping can commence (MARA, 2006). Following this standard, circumference measurements were taken at 100 cm above ground level for all rubber trees both before tapping began and after the trial was concluded. Plants meeting the tapping criteria were identified and marked for three years of long-term observation.

#### 2.4.4 Tapping depth and bark consumption

During each tapping season, 20 trees were randomly sampled to measure their tapping depth and bark consumption. Typically, three to eight sampling surveys are conducted per season, and the average tapping depth per cut and bark consumption per cut are calculated based on the number of tapping cuts. Tapping depth refers to the distance between the inner cut made during tapping and cambium layer. For each rubber tree, the tapping depth was precisely measured three times using a vernier caliper (at the upper, middle, and lower sections of the tapping line), and the average value was calculated. Bark consumption refers to the thickness of the bark removed per cut or over a specific period. Prior to tapping, the initial tapping lines were marked on each tree. After a set interval, the total length was measured using a vernier caliper (taking one measurement each at the upper, middle, and lower sections of the tapping line). Bark consumption per cut was then calculated based on the number of tapping cuts.

#### 2.4.5 Incidence rate and incidence index of tapping panel dryness

Tapping panel dryness (TPD) refers to the condition in which rubber tree latex ducts lose their latex-producing capacity, resulting in the inability to drain latex from all or part of the cut line during tapping. The TPD incidence rate refers to the ratio of infected plants to the total number of plants surveyed. This trial was conducted during the first month after tapping stopped (traditional tapping in December, winter tapping in May). Technical personnel conducted TPD surveys and recorded observations for each rubber tree within each treatment according to the TPD grading standards. The TPD data from all replicate groups were ultimately entered into Excel spreadsheets to calculate the incidence rate and incidence index of TPD. Both the survey and calculation methods strictly followed the technical regulations for exploitation of rubber tree (MARA, 2006).

(1) The TPD grading standards are as follows:

Level 0: No disease
Level 1: length less than 2cm
Level 2: length from 2cm to 1/4 of the tapping line
Level 3: length from 1/4 to 2/4 of the tapping line
Level 4: length from 2/4 to 3/4 of the tapping line
Level 5: length from 3/4 to the entire tapping line

(2) Calculation Methods for the incidence rate and incidence index of TPD:

(5) $$\begin{array}{l} Incidence\;rate\;of\;TPD=Number\;of\;incidence\;plants/\\ Total\;number\;of\;plants\;surveyed\times 100 \end{array}$$

(6) $$\begin{array}{l} Incidence\;index\;of\;TPD=\\ \frac{\sum (Disease\;level\;value\times Number\;of\;plants\;with\;that\;level)}{\left(Most\;severe\;disease\;level\;value\times Total\;number\;of\;plants\;surveyed\right)}\times 100\% \end{array}$$

Additionally, rubber trees exhibiting TPD at level 4 or higher will cease tapping operations and be classified as culled trees. The cessation rate (incidence rate of TPD at levels 4-5) and cessation index (incidence index of TPD at levels 4-5) were calculated based on the incidence rate and incidence index of TPD, respectively. The calculation formula is as follows:

(7) $$\begin{array}{l} Incidence\;rate\;of\;TPD\;at\;levels\;4-5\\ =\frac{Number\;of\;incidence\;plants\;at\;levels\;4-5}{Total\;number\;of\;plants\;surveyed}\times 100\% \end{array}$$

(8) $$\begin{array}{l} Incidence\;index\;of\;TPD\;at\;levels\;4-5=\\ \frac{\left(4\;\times \;number\;of\;plants\;at\;this\;level\;+\;5\;\times \;number\;of\;plants\;at\;this\;level\right)}{Most\;severe\;disease\;level\;value\;\times \;Total\;number\;of\;plants\;surveyed}\times 100\% \end{array}$$

### 2.5 Data statistics and analysis

The collected data underwent basic processing in Excel and were analyzed using SPSS 25.0 software for analysis of variance (ANOVA). The final graphical representations of the data were generated using Origin 2022.

## 3 Results

### 3.1 Comparison of climatic conditions between two rubber tapping seasons

**Figure 1:**
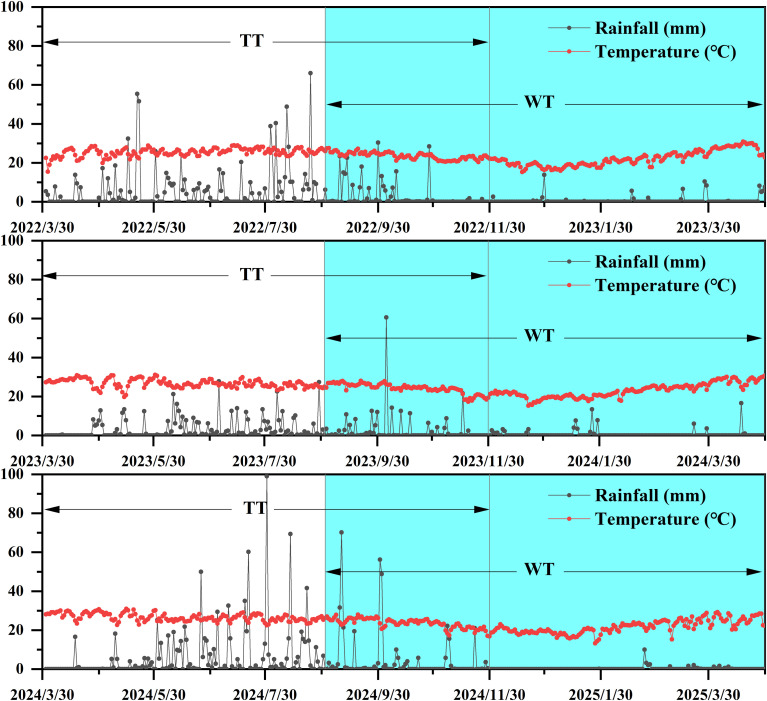
Climate changes during the winter tapping and traditional tapping seasons.

As shown in **Figure 1**, rainfall was predominantly concentrated during the traditional tapping season (April to November), whereas the winter tapping period (September to April of the following year) recorded significantly less precipitation. Specifically, the rainfall during the traditional tapping seasons of 2022, 2023, and 2024 was 1129.83, 683.02, and 1278.61 mm, respectively. In contrast, rainfall during the winter tapping season in the same periods was 332.2, 303.02, and 396.8 mm.

Temperatures during the winter tapping seasons were slightly lower than those during the traditional tapping seasons. The average temperatures during the traditional tapping seasons in 2022, 2023, and 2024 were 24.73, 25.79, and 25.47 °C, respectively, while those during the winter tapping seasons were 22.70, 23.48, and 22.21 °C. Furthermore, the maximum temperatures recorded during the three traditional tapping seasons were 38.81, 40.80, and 40.52 °C, and the minimum temperatures were 11.95, 13.74, and 14.58 °C, respectively. During the winter tapping seasons, the maximum temperatures were 36.69, 37.48, and 36.12 °C, and the minimum temperatures were 10.47, 11.64, and 10.22 °C, respectively.

Over the three-year period, the average rainfall during the winter tapping season was 65.07% lower than that during the traditional tapping season, and the average temperature was 9.99% lower. The average number of rainy days during the winter tapping season was approximately 60 days, compared to approximately 124 days during the traditional tapping season, representing a 51.61% reduction in the average rainy days. Notably, between February and April 2024, only four rainy days occurred, accompanied by a persistent high-temperature period. Compared to the same period in 2023, the number of rainy days in 2024 decreased by 13, the rainfall decreased by 35.8 mm, and the temperature increased by 0.71 °C.

### 3.2 Rubber yield and its composition

As shown in **Table 1**, under the condition of ensuring a certain number of effective tapping trees (with no significant difference), there were no significant differences in fresh latex yield, theoretical dry rubber yield, or yield per unit area between the winter tapping and traditional tapping seasons over the three-year period.

**Table 1:** Rubber yield and its composition under different tapping seasons (calculated using Equations 1–4).

Year	Treatment	Effective tapping trees	Number of tapping	Fresh latex yield (kg)	Theoretical dry rubber yield (kg)	Average dry rubber content (%)	Yield per plant (kg)	Yield per plant per tapping (g)	Yield per unit area (kg/ha)
2022	TT	246	45	1006.00	383.96	38.17	1.56	34.68	450.36
		246	49	1367.00	491.82	35.98	2.00	40.80	651.47
		252	47	1297.00	483.78	37.30	1.92	40.85	580.51
		340	49	1859.00	663.25	35.68	1.95	39.81	670.64
	**Mean**	**271 ± 46.09 A**	**48 ± 2.50 A**	**1382.25 ± 354.19 A**	**505.70 ± 115.93 A**	**36.78 ± 1.16 A**	**1.86 ± 0.20 A**	**39.04 ± 2.94 A**	**588.24 ± 99.76 A**
	WT	212	41	1138.00	403.17	35.43	1.90	46.38	587.14
		285	44	1267.00	455.98	35.99	1.60	36.36	538.56
		259	47	1575.00	566.32	35.96	2.19	46.52	707.89
		271	45	1385.00	483.98	34.94	1.79	39.69	576.17
	**Mean**	**257 ± 31.67 A**	**44 ± 1.91 A**	**1341.25 ± 185.63 A**	**477.36 ± 68.11 A**	**35.58 ± 0.50 A**	**1.87 ± 0.25 A**	**42.24 ± 5.05 A**	**602.44 ± 73.32 A**
2023	TT	266	47	1214.00	473.19	38.98	1.78	37.85	567.83
		254	53	1417.00	551.64	38.93	2.17	40.98	713.33
		289	54	1459.00	588.12	40.31	2.04	37.69	694.63
		345	52	2004.00	790.18	39.43	2.29	44.05	800.86
	**Mean**	**289 ± 40.37 A**	**52 ± 3.11 A**	**1523.50 ± 337.72 A**	**600.78 ± 135.06 A**	**39.41 ± 0.64 A**	**2.07 ± 0.22 A**	**40.14 ± 3.01 A**	**694.16 ± 96.11 A**
	WT	192	54	1350.00	481.50	35.67	2.51	46.44	701.21
		278	54	1249.00	470.87	37.70	1.69	31.37	556.15
		259	53	1462.00	558.48	38.20	2.16	40.69	698.11
		240	53	1431.00	535.51	37.42	2.23	42.10	637.51
	**Mean**	**242 ± 36.92 A**	**54 ± 0.58 A**	**1373.00 ± 95.20 A**	**511.59 ± 42.17 A**	**37.25 ± 1.10 B**	**2.15 ± 0.34 A**	**40.15 ± 6.35 A**	**648.25 ± 68.04 A**
2024	TT	183	44	1097.00	437.92	39.92	2.39	54.39	525.51
		196	46	1263.00	500.15	39.60	2.55	55.47	646.74
		207	47	1381.00	556.96	40.33	2.69	57.25	657.82
		302	42	1842.00	734.41	39.87	2.43	57.90	744.33
	**Mean**	**222 ± 54.23 A**	**45 ± 2.22 B**	**1395.75 ± 319.49 A**	**557.36 ± 127.65 A**	**39.93 ± 0.30 A**	**2.52 ± 0.13 A**	**56.25 ± 1.61 A**	**643.60 ± 90.01 A**
	WT	160	54	1251.00	441.29	35.28	2.76	51.08	642.66
		232	53	1266.00	454.65	35.91	1.96	36.98	536.99
		223	53	1624.00	578.75	35.64	2.60	48.97	723.44
		259	53	1392.50	493.47	35.44	1.91	35.95	587.46
	**Mean**	**219 ± 41.89 A**	**53 ± 0.50 A**	**1383.38 ± 172.51 A**	**492.04 ± 61.90 A**	**35.57 ± 0.27 B**	**2.30 ± 0.44 A**	**43.24 ± 7.89 B**	**622.64 ± 79.86 A**

Different capital letters appearing in the same column for the same year indicate that the differences between treatments are statistically significant at the LSD 0.05 level.

The bold values represent the mean values for each treatment group.

However, due to high temperatures and drought conditions (as described in Section 3.1), the traditional tapping season in 2024 was delayed, resulting in approximately 30 fewer tapping days compared to the winter tapping season. Consequently, the number of tapping in traditional tapping in 2024 was significantly lower than that in winter tapping, while the yield per plant per tapping was significantly higher. Specifically, in 2024, the average number of tapping in traditional tapping was 8 fewer than that in winter tapping, while the average yield per plant per tapping in winter tapping was 23.13% lower than that in traditional tapping.

In addition, the average dry rubber content (DRC) in winter tapping was significantly lower than that in traditional tapping in both 2023 and 2024. Specifically, the DRC in winter tapping was 5.48% lower than that in traditional tapping in 2023, and 10.92% lower in 2024.

### 3.3 Incidence rate and incidence index of tapping panel dryness

**Figure 2:**
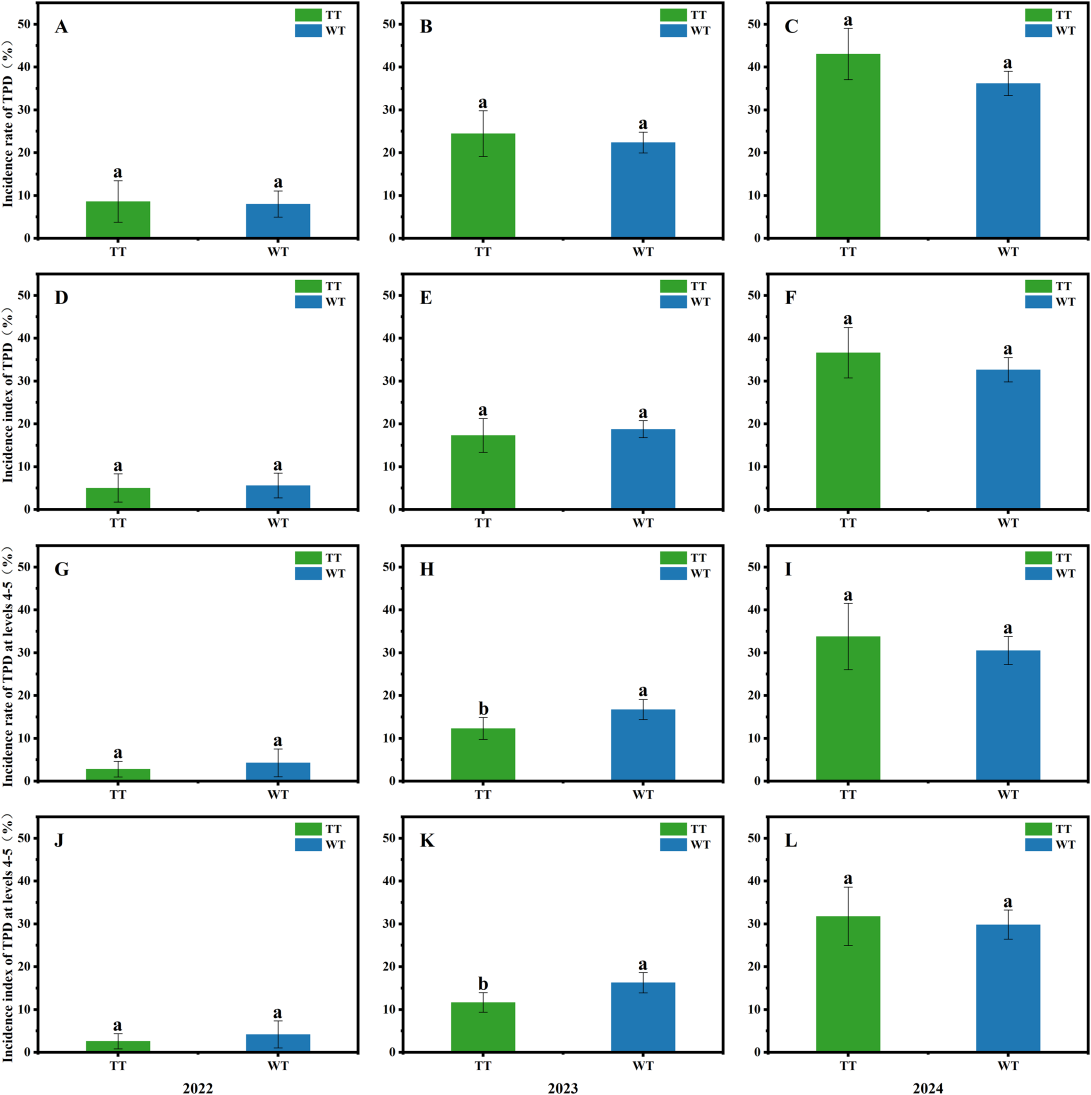
Incidence rate of TPD **(A–C)**, incidence index of TPD **(D–F)**, incidence rate of TPD at levels 4-5 **(G–I)**, and incidence index of TPD at levels 4-5 **(J–L)** in rubber trees under different tapping seasons (calculated using Equations 5–8). Different lowercase letters indicate statistically significant differences between groups (p<0.05); the same lowercase letters indicate no statistically significant differences between groups.

Tapping panel dryness (TPD) significantly affects the number of productive tapping years and yield of rubber trees. As shown in **Figure 2**, no significant differences were observed in either the incidence rate or incidence index of TPD between winter tapping and traditional tapping over the three-year period, indicating that winter tapping has no significant impact on the occurrence of TPD in rubber trees. However, a numerical decrease was observed in both the incidence rate and incidence index of TPD was observed over time with continued winter tapping.

Specifically, the incidence rate of TPD in winter tapping was 15.93% lower than that in traditional tapping in 2024, 8.46% lower in 2023, and 7.11% lower in 2022. Meanwhile, the incidence index of TPD in winter tapping was 10.78% lower than that in traditional tapping in 2024, but was 8.38% higher in 2023 and 11.70% higher in 2022, compared to traditional tapping. A similar trend was observed for the incidence rate and incidence index of TPD at levels 4–5.

Trees affected by TPD at level 4 or higher were classified as culled trees, hence the incidence rate of TPD at levels 4–5 is also termed the culling rate. As shown in **Figure 2**, the incidence rate and incidence index of TPD at levels 4–5 in winter tapping were significantly higher than those in traditional tapping in 2023 by 35.91% and 39.66%, respectively. In contrast, no significant differences were detected in these parameters between the two tapping systems in 2022 and 2024.

### 3.4 Comparison of rubber tapping techniques and post-tapping growth of rubber trees

**Figure 3:**
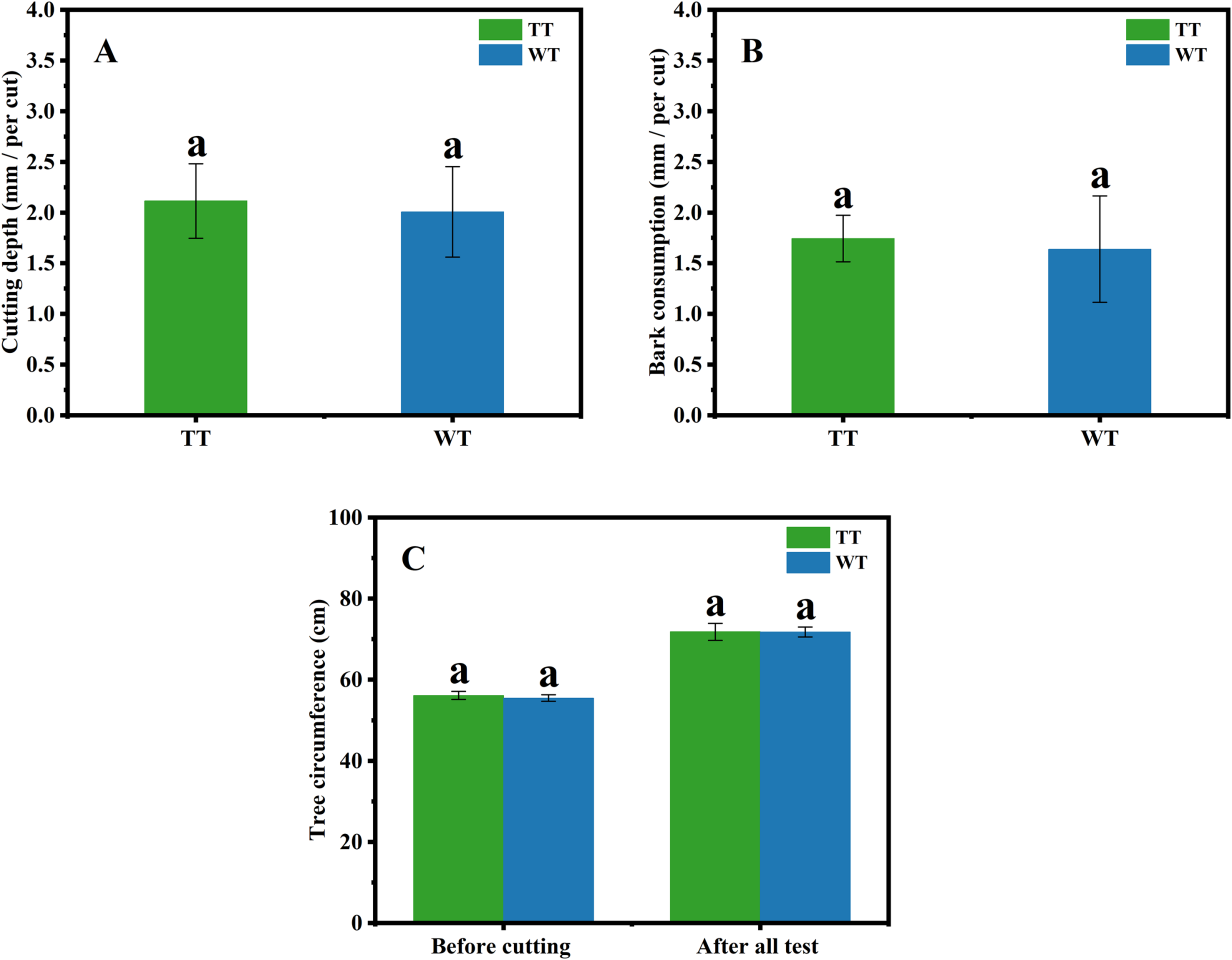
Comparison of tapping depth **(A)**, bark consumption **(B)**, and tree circumference **(C)** in rubber trees between the winter and traditional tapping seasons. Different lowercase letters indicate statistically significant differences between groups (p<0.05); the same lowercase letters indicate no statistically significant differences between groups.

As the quality of tapping techniques significantly affects rubber yield and tree growth, their evaluation is essential to establish and validate the rationality of the experimental design. Severe or destructive tapping practices can lead to a sharp increase in short-term latex production, followed by inhibited tree development, a drastic decline in yield in the following year, and in extreme cases, tree mortality. Therefore, this study evaluated the tapping depth, bark consumption, and tree circumference under winter tapping conditions to determine whether significant differences exist compared to traditional tapping standards. As shown in **Figure 3**, no significant differences were observed in tapping depth, bark consumption, and tree circumference between the two tapping seasons. These results indicate that the winter tapping techniques employed by tappers were largely consistent with those used in traditional tapping. Furthermore, the observed healthy tree growth suggests that no destructive tapping methods were adopted to artificially boost the rubber yield.

### 3.5 Analysis of theoretical economic benefits

The pricing of physical natural rubber trade contracts is rarely determined through simple bilateral negotiations. Instead, it is highly financialized, leading to natural rubber being primarily traded through futures contracts. This analysis is a theoretical assessment based on market fair value. It reveals the potential profit level achievable under ideal market conditions but does not equate to the actual final cash returns received by producers. To evaluate the theoretical economic benefits of winter tapping, we compiled five years of natural rubber futures trading data from the Shanghai Futures Exchange.

**Figure 4:**
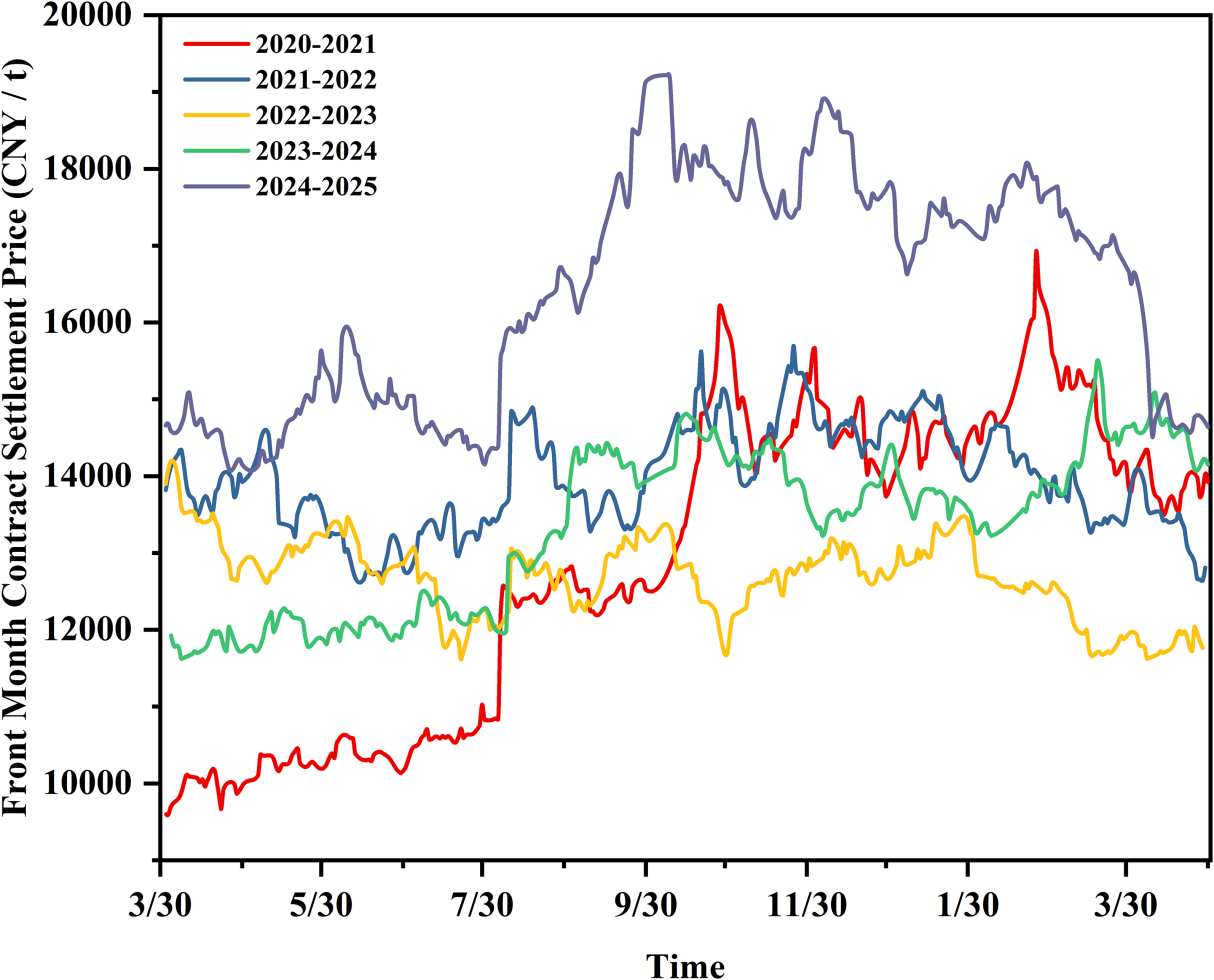
Daily price trend chart for settlement prices of the natural rubber front-month contracts (data sourced from Tushare, https://tushare.pro/). .

As shown in **Figure 4**, the settlement prices of the front-month contracts rose sharply after July 30th in certain years, such as in the 2020–2021, 2023–2024, and 2024–2025 periods, while price fluctuations remained relatively moderate during the 2021–2022 and 2022–2023 periods. The 2024–2025 period was particularly noteworthy, with prices relatively low between April and August 2024, then climbing rapidly from approximately 14,000 CNY/t to approximately 18,000 CNY/t. From October 2024 to April 2025, prices remained high and stable, averaging approximately 17,000 CNY/t. The trend patterns observed in 2020–2021 and 2023–2024 were largely similar to those in 2024-2025.

To compare the economic returns between the two tapping seasons, we categorized the settlement prices of the front-month contracts according to the traditional tapping season (April to November) and winter tapping season (September to April of the following year). It is important to note that the 2021–2023 period coincided with the COVID-19 pandemic, which caused unprecedented global supply chain disruptions, logistical bottlenecks, and demand shocks that temporarily overrode normal seasonal price patterns in natural rubber markets. As shown in **Table 2**, except for 2022-2023, the average settlement price during the winter tapping season was higher than that during the traditional season in all other years. When the atypical 2021–2022 and 2022–2023 periods (which were significantly influenced by non-seasonal factors) are excluded, the average settlement price during winter tapping was 8.86–20.81% higher than that during the traditional season.

**Table 2:** Comparison of settlement prices for natural rubber front-month contracts under different tapping seasons over the past 5 years.

Rubber tapping seasons	Periods	Average settlement price (CNY/t)	Price fluctuation (CNY/t)	Fluctuation ratio (%)
TT	20200401-20201130	11825.37	2460.87	20.81
WT	20200901-20210430	14286.24		
TT	20210401-20211130	13870.12	329.53	2.38
WT	20210901-20220430	14199.65		
TT	20220401-20221130	12750.71	-218.60	-1.71
WT	20220901-20230430	12532.11		
TT	20230401-20231130	12945.65	1146.63	8.86
WT	20230901-20240430	14092.28		
TT	20240401-20241130	15984.88	1653.75	10.35
WT	20240901-20250430	17638.62		

Price fluctuation and fluctuation ratio represent comparisons between winter tapping and traditional tapping within the same year. “-” indicates that the average settlement price in winter tapping was lower than that in traditional tapping.

## 4 Discussion

### 4.1 Validation of the core hypothesis and novelty of winter tapping

This study provides the first comprehensive evidence supporting our central hypothesis that a structured winter tapping system for cold-tolerant clones is technically feasible, agronomically sound, and economically beneficial in China’s marginal rubber-growing regions. The novelty of our approach lies not merely in conducting tapping activities in winter, but in systematically developing and validating an alternative production season that fundamentally challenges the conventional agricultural calendar for rubber cultivation in China. Our findings demonstrate that winter tapping maintains comparable latex yield and tree health while avoiding rainy season disruptions and capturing potential market premiums as suggested by futures price trends, thus confirming the initial hypothesis.

### 4.2 Climate advantages for winter tapping

Climate and environmental factors significantly impact rubber production and tapping operations. Nguyen and Dang (2016) indicated that regions with monthly average temperatures between 25–28 °C are optimal for the cultivation of rubber trees. However, comparatively lower temperatures have been observed to stimulate prolonged latex flow. Specifically, temperatures within the 18–24 °C range promoting higher latex flow rates (Priyadarshan, 2003; Shuochang and Yagang, 1990). Temperatures below 5 °C have been shown to cause cold damage to rubber trees, hindering normal tapping (Cheng et al., 2024; Xu and Pan, 1992). In this study, the average temperature recorded in winter tapping ranged from 22.70 °C to 23.48 °C, slightly lower than that in traditional tapping (24.73 °C to 25.79 °C), yet it remained within an optimal range for latex flow. Concurrently, the minimum temperatures during the winter tapping season were also slightly lower but remained above 5 °C. Therefore, both the average and minimum temperatures during the winter tapping season fell within an appropriate range, which effectively maintained normal tree growth and enabled tapping operations. This finding is particularly significant as it challenges the conventional wisdom that rubber tapping must be confined to warmer months, demonstrating instead that the thermal conditions during Yunnan’s winter are not merely adequate but potentially optimal for latex flow in cold-tolerant clones.

Rao and Vijayakumar (1992) demonstrated that rubber trees thrive in regions with annual rainfall exceeding 2000 mm, characterized by uniform distribution and no distinct dry season, with 125–150 days of overcast or rainy weather annually. Furthermore, the study emphasizes that such stringent rainfall requirements and distribution patterns remain critical factors in rubber production that cannot be overlooked (Korakot et al., 2015). Hazir et al. (2020) found that Malaysia’s dry season occurs from February to March, coinciding with low rubber production, while the high-yield period from September to December largely overlaps with the rainy season. This finding indicates that increased rainfall in Malaysia is more conducive to enhancing rubber yield. However, Mesike and Esekhade (2014) collected and analyzed time-series data on annual average rainfall and total rubber production from 1971 to 2009, revealing a significant inverse relationship between rainfall and rubber yield in Nigeria. This contradiction is probably a consequence of a trade-off between the negative impact of rainfall on tapping operations (e.g., disruption to tapping procedures, reduction in tapper motivation) and its positive effect on tree growth (e.g., promotion of latex synthesis). Therefore, the present study was conducted under controlled conditions to minimize human influence (ensuring consistent tapping systems, tapping frequency, and rubber collection intervals). The results showed that although rainfall during the winter tapping season was significantly lower than that during the traditional season, it did not lead to reduced yields. We hypothesize that lower winter temperatures may reduce surface evaporation and plant transpiration, thereby partially offsetting the effects of reduced precipitation. This helps maintain water balance within the soil-plant system, supporting the normal physiological functions of rubber trees. However, this hypothesis requires validation through monitoring indicators such as soil moisture dynamics and plant water potential, with the aim of confirming it in future research.

### 4.3 The agronomic feasibility of winter tapping

From a yield perspective, winter tapping showed no significant difference from traditional tapping in terms of fresh latex yield, dry rubber yield, and yield per unit area, directly validating our first hypothesis. This indicates that winter tapping is feasible in terms of productivity. However, the winter tapping period coincides with the rubber tree’s leaf shedding and renewal phase. Given the latex production characteristics of the rubber tree, we cannot clearly explain the impact of this process on yield. Therefore, further research is necessary to determine whether leaf shedding and renewal significantly affect yield during winter tapping. Additionally, in 2024, high temperatures and drought reduced the actual operational period for traditional tapping by approximately 30 days. Despite this, latex yield did not show significant differences compared to winter tapping, suggesting that the yield potential of traditional tapping in 2024 was not fully realized. However, these non-human factors led to the comparable yields between the two tapping seasons. Based on these observations, optimizing the timing of winter tapping could further enhance its productivity.

Moreover, the average dry rubber content (DRC) of winter tapping was significantly lower than that of traditional tapping in both 2023 and 2024. This aligns with the findings of Zhang et al. (2025), who reported a characteristic decrease in DRC during the winter dry season. This may be related to lower winter temperatures, reduced physiological metabolic activity due to the rubber tree’s leaf renewal phase, and changes in water balance. More importantly, despite this significant reduction in DRC, dry rubber quality was not adversely affected. Qi et al. (2025) demonstrated that natural rubber from both winter and traditional tapping exhibited consistent properties. Key metrics including plasticity initial (P0), plasticity retention index (PRI), Mooney viscosity, ash content, nitrogen content, and the tensile strength of vulcanized rubber, all met national standards. This finding confirms that while DRC decreases, the fundamental quality parameters remain unaffected, validating the technical viability of winter tapping from a quality perspective. However, latex with a lower DRC implies a higher water content per unit weight, which directly increases the risk of higher transportation costs and processing energy consumption. This may partially offset the potential price advantage gained from winter tapping. A comprehensive cost-benefit analysis incorporating these factors is warranted in future research.

The clone Yunyan 77–4 is prone to impaired latex flow during the hot summer and autumn seasons, often manifesting as temporary cessation of exudation when temperatures are high, with flow resuming only when temperatures drop or humidity increases (Qi et al., 2025; Zhang et al., 2025). This phenomenon, which can be an early sign of or a contributing factor to tapping panel dryness (TPD), indicates a temporary loss of latex production function in the laticifers (Li et al., 2024). It is also a direct reason for the higher incidence rate and incidence index of TPD in traditional tapping compared to winter tapping. However, in 2023 and 2024, the incidence rate and incidence index of TPD at levels 4–5 in winter tapping were significantly higher than those in traditional tapping. This disparity likely stems from two primary factors: 1) a slightly higher proportion of untapped trees in the traditional tapping plots during 2023, and 2) a marginally higher incidence rate of TPD in the first winter tapping season than that in the traditional tapping season. Although the risk of severe TPD in winter tapping did not show a continuously increasing trend within the three-year observation period of this study, this finding must be interpreted with caution. TPD development is a long-term cumulative process, and our three-year study period remains relatively short. Therefore, before promoting winter tapping, longer-term continuous monitoring is essential to fully assess its potential impact on the long-term health and sustainable productivity of rubber trees. Furthermore, future research should delve deeper into the regulatory mechanisms of winter tapping on rubber tree physiology by measuring parameters such as latex flow kinetics, metabolites, and key enzyme activities.

### 4.4 Potential impacts of winter tapping on the rubber industry

The adoption of winter tapping has the potential to influence China’s natural rubber sector and may contribute to supply chain diversification at the regional level.

From a production standpoint, this study confirms that winter tapping maintains annual yield levels while significantly reducing weather-related disruptions caused by rainy seasons, thereby enhancing operational reliability and labor efficiency. This characteristic helps stabilize the domestic rubber supply base and alleviates China’s long-standing production bottlenecks stemming from limited suitable cultivation areas and short tapping seasons. Particularly in major production regions like Yunnan, this approach can optimize the effective tapping period to over eight months, increasing both land productivity and resource use efficiency (Li et al., 2023).

In terms of market mechanisms, winter tapping concentrates output during the traditional tapping off-season (September to April of the following year), coinciding with supply contractions in Southeast Asian major producers due to rainy seasons or natural disasters. The economic analysis confirmed our third hypothesis, as futures price analysis indicates that rubber settlement prices during this period were higher than those of traditional tapping seasons in three out of five years, with premiums ranging from 8.86% to 20.81%. Consequently, winter tapping not only aligns production with a period of historically higher futures prices, suggesting a potential for increased theoretical revenue, thereby boosting their economic returns and production incentives, but also helps address long-standing issues such as labor migration and poor industry sustainability caused by persistently low rubber prices (Liu et al., 2024). This price premium mechanism represents a crucial innovative aspect of winter tapping, transforming a climatic constraint (winter) into an economic advantage. However, the anomalous price relationships observed in 2021–2023 highlight an important caveat: the potential price advantage of winter tapping is predicated on normal market seasonality. Exceptional events that disrupt global supply chains or fundamentally alter demand patterns, such as the COVID-19 pandemic, can temporarily override these seasonal trends. Farmers considering a shift to winter tapping should be aware that while the strategy may offer potential for capturing market premiums under normal conditions, it does not eliminate exposure to broader market risks and extraordinary global events. We have also verified that even when including all five years of data, the average premium across the entire period remains positive (approximately 8.1%), though with higher variability. This supports our conclusion that winter tapping generally aligns with higher-priced periods, while acknowledging the inter-annual variability introduced by exceptional market conditions.

More importantly, at the supply chain level, winter tapping enables China to sustainably export resources during the global rubber supply off-season, partially altering its passive position in international markets. This capability enhances China’s influence in the international pricing system and reduces import risks stemming from fluctuations in Southeast Asian production areas. Particularly amid heightened uncertainties like geopolitical conflicts and climate anomalies, this model provides an additional safeguard for the secure supply of critical strategic materials.

In summary, winter tapping represents a potentially valuable strategic tool for China’s rubber industry to address internal challenges, improve its resilience to market fluctuations, and enhance its position within the global supply chain. Furthermore, it should be noted that this study was conducted exclusively on the cold-tolerant clone “Yunyan 77-4”. This variety is a triploid clone developed through the crossbreeding of GT1 and PR107, exhibiting strong cold tolerance and latex-producing potential. However, it remains unclear whether the conclusions drawn from this research are applicable to other common commercial varieties (such as GT1, PR107, etc.). Future comparative trials of winter tapping across multiple varieties should be conducted in controlled environments or regions with milder winter climates to assess the universality of this strategy across different genetic backgrounds.

## 5 Conclusion

Through a three-year comparative field trial, this study systematically evaluated the production performance and potential value of the cold-resistant rubber tree clone ‘Yunyan 77-4’ under a winter tapping model for sustainable industry development. The results indicate that in rubber-growing regions in northern Yunnan, winter tapping (September to April of the following year) showed no significant differences compared to the traditional tapping (April to November) in terms of latex yield, tapping operation standardization, or tree growth status. This demonstrates the model’s strong technical feasibility. Furthermore, winter tapping optimizes the tapping period, avoids rainy season disruptions, and improves operational efficiency, while the corresponding off-season for natural rubber supply facilitates higher market prices, thereby offering farmers the opportunity to capture potential market premiums, boosting production motivation, and alleviating labor migration issues.

From a broader perspective, the winter tapping model offers an effective pathway for China to overcome geographical and climatic constraints, expand suitable tapping areas, and optimize existing production systems. This not only enhances domestic self-sufficiency in natural rubber and reduces reliance on imports but also holds strategic importance for enhancing domestic supply security and reducing reliance on imports. To further advance the industrial application of this model, future research should focus on integrating and promoting technologies such as cold-resistant variety breeding, standardized tapping techniques, tree nutrition management, and pest and disease control. This will enable large-scale, high-quality winter tapping development, ultimately driving China’s natural rubber industry toward sustainable, efficient, and secure transformation.

## Data Availability

The original contributions presented in the study are included in the article/supplementary material. Further inquiries can be directed to the corresponding author.
